# THERAPEUTIC APPROACH OF COMPLICATED HYDATID DISEASE: ROLE OF ENDOSCOPIC RETROGRADE CHOLANGIOPANCREATOGRAPHY IN CHOLANGIOHYDATIDOSIS

**DOI:** 10.1590/0102-672020220002e1699

**Published:** 2023-01-09

**Authors:** Franz GONZÁLEZ-ARBOLEDA, Felipe PIZARRO, Cristian LINDNER, Fermín CAQUEO

**Affiliations:** 1 Regional Hospital, Digestive Surgery Department – Talca, Chile;; 2 Catholic University of Maule, Faculty of Medicine – Talca, Chile;; 3 Regional Hospital, Radiology Department – Talca, Chile.

**Keywords:** Echinococcus, Echinococcosis, Bile Duct Diseases, Cholangiopancreatography, Endoscopic Retrograde, Echinococcus, Equinococose, Doenças dos Ductos Biliares, Colangiopancreatografia Retrógrada Endoscópica

## Abstract

**BACKGROUND:**

Hydatid disease, a parasitic infestation caused by *Echinococcus granulosus* larvae, is an infectious disease endemic in different areas, such as India, Australia, and South America. The liver is well known as the organ most commonly affected by hydatid disease and may present a wide variety of complications such as hepatothoracic hydatid transit, cyst superinfection, intra-abdominal dissemination, and communication of the biliary cyst with extravasation of parasitic material into the bile duct, also called cholangiohydatidosis. Humans are considered an intermediate host, exposed to these larvae by hand-to-mouth contamination of the feces of infected dogs.

**AIM:**

This study aimed to highlight the role of endoscopic retrograde cholangiopancreatography in patients with acute cholangitis secondary to cholangiohydatidosis.

**METHODS:**

Considering the imaging findings in a 36-year-old female patient with computed tomography and magnetic resonance imaging showing a complex cystic lesion in liver segment VI, with multiple internal vesicles and a wall defect cyst that communicates with the intrahepatic biliary tree, endoscopic biliary drainage was performed by endoscopic retrograde cholangiopancreatography with papillotomy, leading to the discharge of multiple obstructive cysts and hydatid sand from the main bile duct.

**RESULTS:**

Clinical and laboratory findings improved after drainage, with hospital discharge under oral antiparasitic treatment before complete surgical resection of the hepatic hydatid cyst.

**CONCLUSIONS:**

Endoscopic retrograde cholangiopancreatography is a safe and useful method for the treatment of biliary complications of hepatic hydatid disease and should be considered the first-line procedure for biliary drainage in cases of cholangiohydatid disease involving secondary acute cholangitis.

## RESUMO

**RACIONAL:**

A doença hidática, uma infestação parasitária causada pelas larvas de *Echinococcus granulosus*, é uma doença infecciosa endêmica em diferentes áreas como Índia, Austrália e América do Sul. O fígado é conhecido como o órgão mais comumente afetado pela hidatidose, podendo apresentar uma grande variedade de complicações como trânsito hidático hepato-torácico, superinfecção do cisto, disseminação intra-abdominal e comunicação do cisto biliar com extravasamento de material parasitário para o ducto biliar ou também chamada de colangio-hidatidose O ser humano é considerado um hospedeiro intermediário, exposto a essas larvas pela contaminação mão-boca das fezes de cães infectados.

**OBJETIVO:**

Destacar o papel da endoscópica por colangiopancreatografia retrógrada em pacientes com colangite aguda secundária à colangio-hidatidose.

**MÉTODOS:**

Considerando os achados de imagem, em paciente feminina de 36 anos de idade, com imagens de tomografia computadorizada e ressonância magnética mostrando uma lesão cística complexa no segmento hepático VI, com múltiplas vesículas internas e um defeito de parede cística que se comunica com a árvore biliar intra-hepática foi realizada drenagem biliar endoscópica por colangiopancreatografia retrógrada com papilotomia, levando à descarga de múltiplos cistos obstrutivos e areia hidática da via biliar principal.

**RESULTADOS:**

Os achados clínicos e laboratoriais melhoraram após a drenagem, com alta hospitalar sob tratamento antiparasitário oral antes da ressecção cirúrgica completa do cisto hidático hepático.

**CONCLUSÕES:**

A endoscópica por colangiopancreatografia retrógrada é um método seguro e útil para o tratamento das complicações biliares da hidatidose hepática, devendo ser considerado o procedimento de primeira linha para drenagem biliar nos casos de colangio-hidatidose que envolve colangite aguda secundária.

## INTRODUCTION

Hydatid disease, a parasitic infestation caused by the larvae of *Echinococcus granulosus*, is an endemic infectious disease in different areas, such as India, Australia, and South America^8,16^. The human is considered an intermediated host, exposed to these larvae by hand-to-mouth contamination from stool from infected dogs^16^. The liver is well-known as the most common organ affected by hydatid disease, which may present a wide variety of complications such as hepatothoracic hydatid transit, cyst superinfection, intra-abdominal dissemination, and biliary cyst communication with parasitic material leak into the bile duct, also called cholangiohydatidosis^3,14^. The migration of parasitic elements into the bile ducts throughout the cystic communication may generate a biliary obstructive syndrome with secondary acute cholangitis^7^. Although there are different biliary drainage techniques, endoscopic retrograde cholangiopancreatography (ERCP) is actually considered the first-line drainage procedure in biliary obstruction syndrome, mainly due to its high effectiveness and lower risk of adverse events^4,6,9^. In this article, we aim to highlight the role of ERCP in patients with acute cholangitis secondary to cholangiohydatidosis.

### Case Report

A 36-year-old woman with a medical history of bronchial asthma presented to our hospital with the complaints of generalized and diffuse upper abdominal pain, fever, and progressive jaundice of 2-day duration. On hospital admission, she was mild febrile and tachycardic. Initial evaluation revealed a total bilirubin concentration (2.78 μmol/L), elevated white blood cell count (WBC) (10.980/mm^3^), and C-reactive protein (CRP) (118 μmol/L). Alanine aminotransferase (ALT), aspartate aminotransferase (AST), creatinine, platelet count, and prothrombin time were normal. After the severity assessment, general supportive care was immediately started, with clinical improvement after completing antimicrobial therapy.

**Figure 1:**
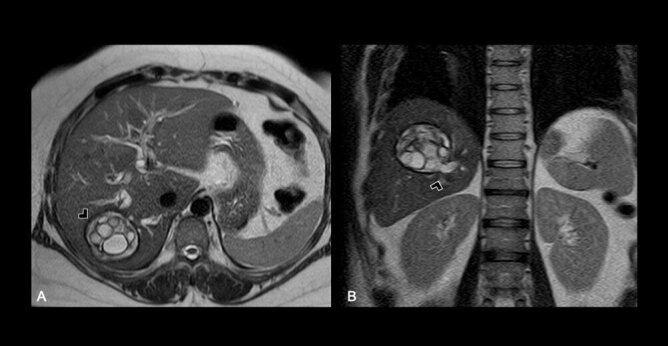
(A) Axial. Complex cystic lesion in the VI hepatic segment, with multiple inner vesicles and diffuse wall thickening; (B) Coronal. Cystic communication with the intrahepatic biliary tree.

**Figure 2:**
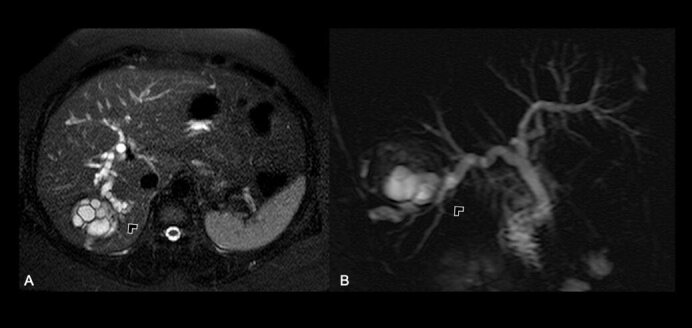
Biliary tree communication. (A) Axial fat-saturated T2-weighted image; (B) Heavily T2-weighted image sequence.

**Figure 3:**
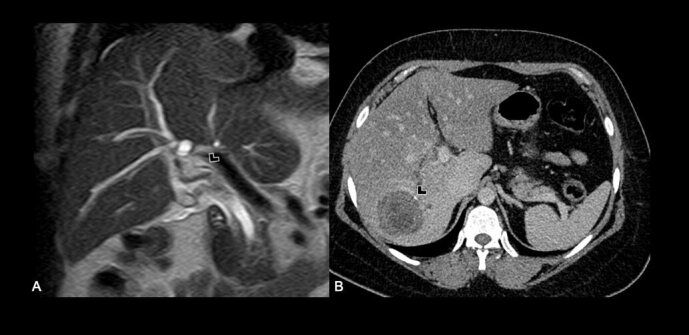
(A) Intrahepatic and extrahepatic bile duct dilatation with endoluminal hydatid sand; Coronal T2-weighted image; (B) Complex cystic lesion with isolated peripheral calcifications; Axial contrast – enhanced computed tomography.

In view of her favorable clinical response, computed tomography (CT) and magnetic resonance imaging (MRI) were obtained, depicting a complex cystic lesion in the VI hepatic segment, with multiple inner vesicles (Figure 1), and a cystic wall defect that communicates to the intrahepatic biliary tree (Figure 2), associated with an intrahepatic and extrahepatic bile duct dilatation (Figure 3).

**Figure 4:**
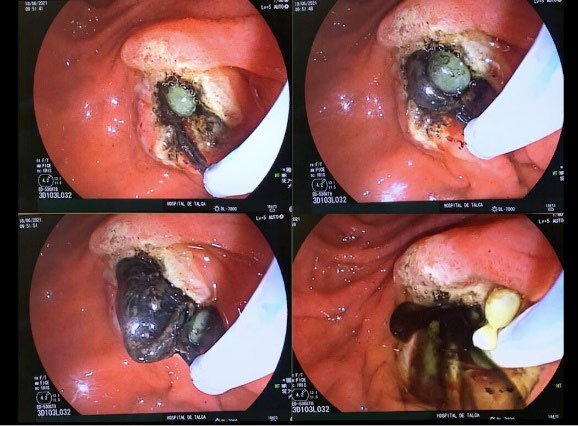
Endoscopic retrograde cholangiopancreatography showing hydatid material extraction from the bile duct.

Considering the imaging findings, endoscopic biliary drainage by ERCP with papillotomy was performed, leading to the discharge of multiple obstructive daughter cysts and hydatid sand from the main biliary tract (Figure 4). Both clinical and laboratory findings improved after drainage, with the patient being discharged home under oral antiparasitic treatment prior to its complete surgical hepatic hydatid cyst resection. Written informed consent was obtained from the patient for publication of this case and any accompanying images (CEC-01 Servicio de Salud del Maule Scientific Ethics Committee nº 61606900-4).

## DISCUSSION

Hepatic hydatidosis is a complex zoonotic disease that remains a major worldwide health problem, especially in endemic countries, with an estimated annual incidence of above 50 cases per 100,000 people^1^. At present, a wide variety of complications have been described^12^. However, the cystic communication to the bile duct with parasitic material migration into the biliary tree represents a life-threatening condition that can lead to mild-to-severe acute cholangitis, secondary pancreatitis, sepsis, and death^3^. Clinically, these patients present symptoms of biliary obstruction syndrome, while laboratory findings are nonspecific^7^. Imaging findings (abdominal ultrasound, CT, or MRI) can depict a communication between a cystic wall and biliary radicles, with the existence of pus and linear structures passing through the defect and filling the bile tract^12^. According to the initial severity assessment, general supportive care and early intravenous antimicrobial therapy are key elements in the first-line therapy of cholangiohydatidosis, especially among those cases that involve bile duct infection and secondary biliary sepsis^11^. The biliary tract drainage is considered the main treatment, which can be performed by endoscopic management, conventional surgery, or percutaneous transhepatic biliary drainage (PTBD)^10^. Compelling evidence supports that ERCP is the procedure of choice for biliary decompression and drainage for biliary obstruction, including uncommon causes such as cholangiohydatidosis^7,4,11,15^.

Despite the risk of post-ERCP complications, which ranges between 0.5 and 5%^2^, clinical evidence shows that ERCP drainage has both high success rates (80–100%)^5,13^ and lower adverse outcomes such as bile leakage, inflammation, pain, and impairment of the patient’s quality of life, compared to PTBD and conventional surgery^7,4,13,15^.

## CONCLUSIONS

The ERCP is a safe and helpful method for treating biliary complications of hepatic hydatidosis and should be considered the first-line procedure for biliary drainage in cases of cholangiohydatidosis that involves secondary acute cholangitis.
